# Strategies of Speech Interaction between Adults and Preschool Children with Typical and Atypical Development

**DOI:** 10.3390/bs9120159

**Published:** 2019-12-16

**Authors:** Elena Lyakso, Olga Frolova, Aleksey Grigorev, Viktor Gorodnyi, Aleksandr Nikolaev

**Affiliations:** Department of Higher Nervous Activity and Psychophysiology, Saint Petersburg State University, Universitetskaya emb. 7-9, 199034 Saint-Petersburg, Russia; olchel@yandex.ru (O.F.); a.s.grigoriev89@gmail.com (A.G.); wimndgor@mail.ru (V.G.); al.nikolajew@gmail.com (A.N.)

**Keywords:** strategies of speech interaction, “mother–child” dyads, autism spectrum disorders, Down syndrome, orphans

## Abstract

The goal of this research is to study the speech strategies of adults’ interactions with 4–7-year-old children. The participants are “mother–child” dyads with typically developing (TD, *n* = 40) children, children with autism spectrum disorders (ASDs, *n* = 20), Down syndrome (DS, *n* = 10), and “experimenter–orphan” pairs (*n* = 20). Spectrographic, linguistic, phonetic, and perceptual analyses (*n* = 465 listeners) of children’s speech and mothers’ speech (MS) are executed. The analysis of audio records by listeners (*n* = 10) and the elements of nonverbal behavior on the basis of video records by experts (*n* = 5) are made. Differences in the speech behavior strategies of mothers during interactions with TD children, children with ASD, and children with DS are revealed. The different strategies of “mother–child” interactions depending on the severity of the child’s developmental disorders and the child’s age are described. The same features of MS addressed to TD children with low levels of speech formation are used in MS directed to children with atypical development. The acoustic features of MS correlated with a high level of TD child speech development do not lead to a similar correlation in dyads with ASD and DS children. The perceptual and phonetic features of the speech of children of all groups are described.

## 1. Introduction

Features of mothers’ speech (MS) addressed to young children have been studied during the last few decades. The features of MS directed to children in the first year of life and MS functions have been described in the materials of different languages. The differences between MS and speech addressed to adults in regards to temporal and prosodic characteristics have been revealed, allowing the consideration of MS as a unique phenomenon [1,2,3]. Despite controversial views on MS, the researchers indicated the importance of MS addressed to infants for interaction and emotional contact in the “mother–child” dyad and the identification of the mother by the child via voice [4]. The child’s specific features and the type of child’s pathology affect the mother’s emotional reactions and her speech [5]. Parents of children with intellectual disabilities adapt to the level of intellectual and linguistic development of their children, facilitating the development of communication skills and attention [6]. Mothers and fathers of children with Down syndrome (DS) use more emotional speech [7], more directive utterances [8], and ask fewer questions than parents of typically developing (TD) peers. Authors [6] supposed that parents of DS children use speech appropriated to TD children of lower mental ages. Mothers of children with autism spectrum disorders (ASDs), like mothers of TD children, correct their speech depending on the linguistic skills of their children. Mothers of ASD children with high levels of verbal skill use more questions and support the speech behavior of the child. Mothers of ASD children with worse verbal skills use more commands and support the motor activity of children [9]. Mothers of TD, ASD, and DS Italian children of the same mental age (24 months) were compared. It was revealed that mothers of DS children use emotional speech more often than mothers of other children. Mothers of ASD children refer to the child by name and talk about themselves more often than mothers of TD children. Authors considered this finding as the result of ASD children’s deficit in engaging in social interactions [10]. The studies of adults’ (parents, experimenter) interactions with children of preschool age are few [11]. The development of children with maternal deprivation displays some features in cognitive, emotional, and speech aspects caused by interactions with a limited number of adults (caregivers and the staff of the institution) [12,13].

The goal of our study is to reveal the correlations of the speech strategy of mothers’ interactions with typically and atypically developing 4–7-year-old children and the features of children’s speech.

## 2. Materials and Methods

### 2.1. Data Collection

The participants were “mother–child” dyads with 4–7-year-old TD children (*n* = 40), children with ASD (*n* = 20), DS (*n* = 10), and “experimenter–orphan” pairs (*n* = 20) with children without genetic syndromes and severe neurological disorders (orphans with mild intellectual disabilities [ID] and mixed specific developmental disorders [DD]). All of the children were born and living in Saint Petersburg in monolingual environments (the parents of the children were born in Saint Petersburg or have been living there for more than ten years before infants’ births). For all children, the data on development was obtained from the moment of birth. For children with ASD additionally – scores on questionnaires, Child Autism Rating Scale (CARS) score [14], and diagnoses were obtained. For all mothers, information about age, education, presence/absence of diseases, and family were obtained. The places of audio and video recording were at home, in the laboratory, and in the orphanage. The situations of speech recording were play with a standard set of toys, dialogues with the mother (experimenter for orphans), and picture description together with the mother. The behavior of the experimenter was standardized during interaction with all orphans. The recordings were made by the “Marantz PMD660” recorder with a “SENNHEIZER e835S” external microphone and “SONY HDR-CX560E” camera.

### 2.2. Data Analysis

The design of the study included: expert analysis of the audio fragments of mothers’ and children’s speech during the interaction; analysis of the nonverbal behavior of the mother and the child in the process of the interaction; perceptual, acoustic, phonetic, and statistical analyses of MS directed to children; perceptual and phonetic analyses of child’s words.

Expert analysis of the audio fragments of speech interactions in “mother–child” dyads (*n* = 10 experts, aged 31.3 ± 9.66 years) and “experimenter–child” pairs (*n* = five experts, aged 27 ± 5.43 years) was conducted. The duration of the fragments (from four to 30 fragments for each dyad/pair) was individual. The listening time was 40 h. The criterion for choosing fragments was the verbal and/or voice attraction of attention by the mother/experimenter or the child of one’s partner. One expert made a selection of fragments from an audio recording. Each fragment was listened to by 10 experts for children from families and five experts for orphans. We estimated 21 elements of maternal speech behavior and 19 indicators of child speech level.

Expert analysis of nonverbal behavior in “mother–child” dyads/“experimenter–child” pairs on the basis of video records was conducted. Video tests containing fragments of the interaction in the dyads/pairs (*n* = 51) were created by two experts. The duration of each fragment was one minute. Expert analysis was performed by five experts (aged 33.2 ± 6.8 years) with professional experience of working with children. The experts watched the video fragments without audio.

Perception, acoustic, and phonetic features of MS addressed to children: The aim of the perceptual study was the review of listeners’ recognition of the function of MS and the mother’s emotional state. The test sequences included: MS directed to children (*n* = three tests, for 10 samples of the ASD test, 10 samples of the DS test, and for 10 samples of the TD test). Perceptual analysis of child’s speech: four test sequences were created. The test sequences included the words of TD, ASD, DS children, and orphans (for 30 words of the ASD test, for 30 words of the DS test, for 30 words of the TD test, and for 30 words of the orphans). The listeners had to recognize the meaning of the child’s words via speech.

The listeners were Russian-speaking adults (*n* = 465, aged 16–82 years, 26.7 ± 16.6 years). The factor of the adult’s experience of interaction with children (at the household level) was not significant, so all the data are presented together.

Spectrographic analysis of mother’s speech was carried out in the “Cool Edit Pro” sound editor. The duration of maternal words and phrases, the pauses between words in the phrase, and between phrases in the utterance were determined. We analyzed and compared pitch values, max and min values of pitch, pitch range, formant frequency (F1, F2), duration of vowels, and the stationary part of vowels from mothers’ words addressed to TD, ASD, and DS children. Vowel formant triangle areas [15] and the vowel articulation index [16] were calculated. Phonetic transcription of children’s words was conducted on the basis of Speech Assessment Methods Phonetic Alphabet (SAMPA) for the Russian language. Maternal speech was described using syntagmatic transcription.

All procedures were approved by the Health and Human Research Ethics Committee (HHS, IRB 00003875, St. Petersburg State University) and written informed consent was obtained from parents (official curator for orphans) of the child participant.

## 3. Results

### 3.1. Speech Interaction in “Mother–Child” Dyads: Expert Analysis of the Audio Fragments of Speech Samples

It was shown that mothers of TD children and children with ASD and DS used different strategies of speech interaction with their child. Progress in the speech development of TD children correlated with MS features: if the mother spoke clearly, F (19.270) = 234.42, *p* < 0.0001, R^2^ = 0.943, the child spoke clearly (Beta = 0.150) and used phrases (Beta = 0.3297); if the mother encouraged the child, F (19.270) = 16.667, *p* < 0.0001, R^2^ = 0.540, and asked questions, F (19.270) = 208.41, *p* < 0.0001, R^2^ = 0.936, the child answered the mother’s questions (Beta = 0.584), asked questions (Beta = 0.160), and used phrases (Beta = 0.154); if the mother made pauses between phrases, F (19.270) = 40.477, *p* < 0.0001, R^2^ = 0.740, the child used phrases (Beta = 0.276) and asked questions (Beta = 0.100)—Multiple Regression analysis.

The repetition of the words spoken by the TD children by the mothers, F (19.270) = 35.568, *p* < 0.0001, R^2^ = 0.715; the highlighting of certain words, F (19.270) = 26.909, *p* < 0.0001, R^2^ = 0.654; and the stretching of sounds in words, F (19.270) = 5.625, *p* < 0.0001, R^2^ = 0.233, were due to the low levels of speech of the TD children—the speech was indistinct (Beta = 0.356), the replies were “yes-no” (Beta = 0.127), and the child did not answer the mother’s questions and did not ask questions.

The initiative manifested by the mother in interacting with the ASD child correlated with the following characteristics of her speech: emotional (0.56 Spearman correlation, *p* < 0.05), the mother spoke loudly (0.78), clearly (0.94), addressed the child (0.91), referred to the child by name (0.82), asked questions (0.71), encouraged the child (0.55), instructed the child (0.76), repeated the question or the same words (0.87), repeated the words for the child (0.62), specified what the child said (0.60), grammatically simplified (0.91), singled out individual words (0.70), stretched sounds in words (0.58), and paused between phrases (0.60). These features of MS, F (33.96) = 34.131, *p* < 0.0001, R^2^ = 0.921, correlated with the characteristics of the ASD child’s speech: the child’s speech was emotional (Beta = −0.157, *p* < 0.009), the child answered the mother’s questions (Beta = 0.390, *p* < 0.003), responded to the mother’s reply (Beta = 0.390, *p* < 0.003), used a replica of the word (Beta = 0.220, *p* < 0.03), and used a “yes-no” replica (Beta = 0.209). MS addressed to ASD children was characterized by the repetition of children’s words, the clarification of children’s utterances, the simplification of speech, the emphasis of words by the voice, the stretching of sounds in words, and the presence of long pauses between phrases. However, these features of MS do not always lead to progress in the development of the ASD child’s speech.

Direct correlations between the characteristics of MS and the features of DS child speech behavior were shown: if the mother spoke loudly, the child used a loud voice, F (1.58) = 10.101, *p* < 0.002 (R^2^ = 0.148; Beta = 0.385); the mother’s emotional speech correlated with emotional child speech, F (1.58) = 0.425, *p* < 0.02 (R^2^ = 0.086; Beta = 0.292); if the mother demonstrated joy, the child was happy, F (1.58) = 34.511, *p* < 0.0001 (R^2^ = 0.373; Beta = 0.610)—Regression analysis; if the mother was angry, the child was also angry, F (3.53) = 518, *p* < 0.0001 (R^2^ = 0.593; Beta = 0.660). However, a clear articulation of the MS words, F (3.56) = 45.607, *p* < 0.0001, R^2^ = 0.710, was correlated both with clear pronunciation of the words by the child (Beta = 0.653) and with fuzzy articulation of the child (Beta = 0.769).

Though the behavior and speech of the experimenter were standardized, if the adult highlighted certain words, F (1.48) = 6.654, *p* < 0.01 (R^2^ = 0.122; Beta = 0.349), or stretched sounds in the words, F (1.48) = 9.84, *p* < 0.01 (R^2^ = 0.17; Beta = 0.12), the number of DD children’s answers increased. The number of ID children’s answers increased if the adult used emotional speech, F (1.48) = 6.49, *p* < 0.01 (R^2^ = 0.119; Beta = 0.345).

### 3.2. Elements of Nonverbal Behavior of Mothers and Children in the Process of Interaction: Expert Analysis

The mothers of the TD children, children with ASD, and children with DS often looked at the child (94% of the answers of experts for the mothers of TD children, 99% for mothers of ASD and DS children) and rarely showed discontent (19%—DS, 7%—TD, and 3%—ASD). The mothers of all groups of children attracted the attention of the child with eyes (44%—TD, 43%—ASD, and 40%—DS) and used gestures (56%, 60%, and 56%). Mothers of children with ASD and with DS were more likely to touch children in the process of interaction (93%—ASD and 80%—DS) compared to mothers of TD children (30%) and to attract the child’s attention more through gestures (66%—ASD and 57%—DS) than mothers of TD children (41%). Mothers of DS children were more often dissatisfied with their children (19%) than mothers of ASD children (3%) and TD children (7%), smiled less (36%) than mothers of ASD children (50%) and TD children (51%), and looked around less often (14%) than mothers of ASD children (51%) and TD children (48%).

The nonverbal behavior of ASD children and children with DS differed from the behavior of TD children by a large number of behavioral elements: TD children smiled more often (56%) than ASD children (19%) and DS children (33%), others showed discontent (1%—TD children, 19%—ASD children, and 47%—children with DS), looked at the mother more often (71%), used “eye-to-eye” contact (56%), and looked at the object (85%). Children with ASD were more likely to look around (91%) than children with DS (76%) and TD children (62%) and to touch mothers more often (51%) than children with DS (44%) and TD children (16%).

Orphans with DD and ID smiled when they interacted with the experimenter (60%—DD children and 68%—ID children). Children with ID looked at the adult more often than children with DD (64%—DD children and 75%—ID children), but they rarely looked at the object compared to DD children.

### 3.3. Perception, Acoustic, and Phonetic Features of MS Addressed to the Child

#### 3.3.1. Perception Data

**Figure 1:**
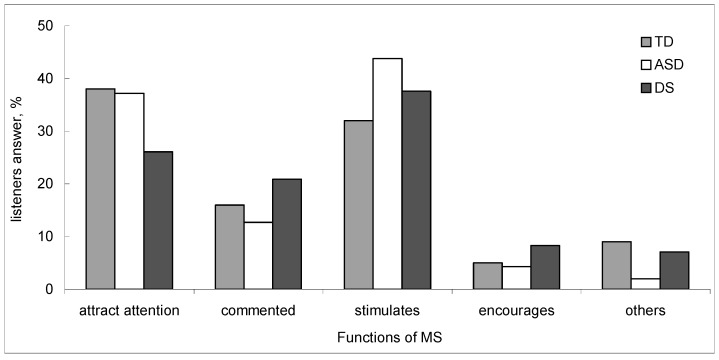
Functions of mother’s speech are estimated by adults on the basis of listening to test sequences containing MS addressed to children. Vertical axis—listener’s answer, %.

MS directed to TD children and children with DS (38% and 37.2%) was aimed at attracting the attention of the child more than MS addressed to ASD children (Figure 1).

Mothers of DS children commented more on the child’s utterances (20.9%) than mothers of ASD children (12.7%) and TD children (16%). MS addressed to ASD and DS children stimulated children to verbal response more (43.8% and 37.6% ASD and DS, respectively) than MS addressed to TD children (32%) with already well-formed speech. MS addressed to TD children and children with ASD contained equally few encouragements for the child (5% and 4.3%), whereas mothers of DS children encouraged their children more often (8.3%).

Determining the emotional state of the mothers, the listeners noted a calm state for mothers of TD, ASD, and DS children, but the number of listeners’ answers was minimal for mothers of DS children (32.2%—DS, 64%—TD, and 52.2%—ASD). According to the responses of listeners, the mothers of ASD children manifested a state of joy more frequently (19.7%) than mothers of DS children (17.9%) and TD children (15%). The listeners noted the states of anger (23.5%—DS, 10.5%—ASD, and 6%—TD), sadness (10.2%—DS, 7.8%—ASD, and 7%—TD), and aggression (10.1%—DS, 7.2%—ASD, and 2%—TD) for mothers of DS children more often than for mothers of ASD and TD children.

#### 3.3.2. Acoustic Data

**Figure 2:**
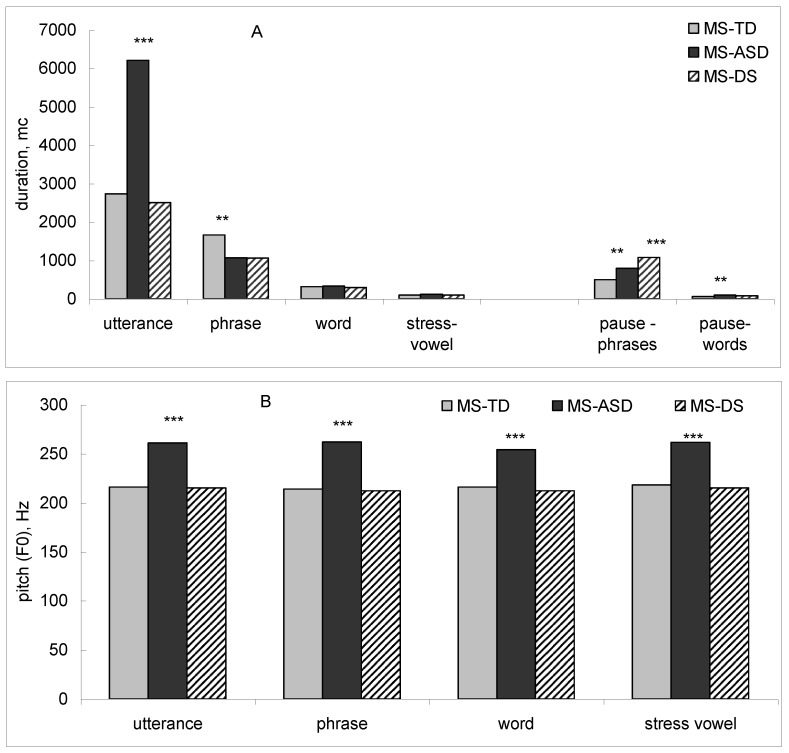
Acoustic features of MS addressed to typically developing children, children with autism spectrum disorders, and children with Down syndrome: (**A**) Duration; (**B**) Pitch values. ** *p* < 0.005, *** *p* < 0.001—Mann–Whitney test.

The mother’s utterances addressed to ASD children were longer (*p* < 0.001—the Mann–Whitney criterion) than the utterances directed to TD children and children with DS, and contained shorter phrases (*p* < 0.005). In MS addressed to children with DS, the duration of pauses between phrases in utterances was longer than the MS directed to TD children (*p* < 0.005). The pauses between phrases (*p* < 0.005) and between words (*p* < 0.005) were longer in MS addressed to ASD than to TD children (Figure 2A).

Pitch values were significantly higher (*p* < 0.001—Mann–Whitney test) in MS addressed to ASD than MS addressed to TD children and children with DS in utterances, phrases, words, and stressed vowels (Figure 2B).

The pitch values of the voices of ASD children’s mothers were higher if the child was older, F (1.74) = 4.531, *p* < 0.03 (R^2^ = 0,057; Beta = 0.240)—Regression analysis. Mothers’ voices addressed to ASD boys were higher than those directed to ASD girls, F (1.74) = 4.118, *p* < 0.04 (R^2^ = 0.054; Beta = 0.231). The lower the voice of the ASD child’s mother (the speech was less emotional) was, the higher the child’s CARS score, F (1.74) = 37.397, *p* < 0.0001 (R^2^ = 0.336; Beta = −0.579).

Discriminant analysis showed the difference in pitch values of MS as a function of the gender of the TD child, F (6.47) = 2.879, *p* < 0.01 Wilks’ Lambda—0.731 Wilks’ = 0.800. Mothers’ communications with TD boys were more emotional (the pitch values were higher) than with the girls, F (6.47) = 2.872, *p* < 0.01 (R^2^ = 0.268; Beta = −1.815).

For MS addressed to DS children, the correlation, F (1.21) = 5.790, *p* < 0.002 (R^2^ = 0.216; Beta = −0.465—Regression analysis), between the child’s age and the pitch values of the mother’s phrases was revealed. The mother’s voice was higher if the child was younger. The age and the gender of DS children were not correlated with the speech features of their mothers.

The maximal values of stressed vowel formant triangle areas (341,498 conv. units) and the minimal values of unstressed vowel formant triangle areas (135,067.8 conv. units) were revealed in MS addressed to children with DS. The values of stressed vowel formant triangle areas in MS addressed to children with ASD (180,670 conv. units) were higher than values in MS addressed to TD children (177,800.5 conv. units). The values of vowel articulation index (VAI) for stressed vowels in words from MS directed to children with DS (1.16) were higher than the values of VAI for stressed vowels for MS directed to children with ASD (0.95) and TD children (0.93).

The data on the values of the vowel formant triangle areas and the values of VAI indicated clearer articulation in MS addressed to children with speech disorders, i.e., ASD and DS, than to TD children. The articulation of the mothers of DS children was clearer than the articulation of the mothers of TD and ASD children.

#### 3.3.3. Phonetic Data

MS addressed to ASD children contained fewer reduced vowel phonemes (up to six reduced phonemes in the utterance, at an average of 3.4) compared to MS directed to TD children (up to nine reduced phonemes, with a mean of 4.5). The results of the phonetic analysis indicated the use of a pronunciation standard for the Russian language by mothers of DS children. The number of reduced phonemes in the utterances of mothers of DS children was up to 28% of the total number of phonemes used in speech.

### 3.4. Perception Data of Child Speech

Listeners recognized correctly 65.8% of the words of TD children, 44.8% of the words of ASD children, and 17.1% of the words of DS children in the tests, as well as 43% of orphans’ words. The amount of words unrecognized by listeners was significantly more (*p* < 0.001) for DS children than for ASD and TD children (*p* < 0.005), and for ASD children than for TD children.

### 3.5. Phonetic Data of Child Speech

TD, ASD, DS children, and orphans used all the vowel phonemes of the Russian language. The consonant phonemes differed in TD, DS, and ASD children in the frequency of occurrence of the phoneme /r/. In ASD child speech, the phoneme /R/ occurred (a uvular trembling). For ASD child speech, there were replacements of phonemes: /s’/ to S/, /t’/ to /tS’/, and /j/ to /r’/, the non-formation of phonemes: /r/, /tS’/, /S/, and /Z/, and the use of vocalized /r’/ and aspirated /k/. In contrast to children with ASD and TD children, children with DS did not have the phonemes /b’/, /m’/, /f/, /z/, /z’/, /r’/, /S/, /S’/, and /r/. Thus, the lack of formation of the majority of consonant phonemes in DS children was shown, which leads to a lack of speech in children. The omissions of phoneme /r/ and the substitutions of phonemes /r/ to /R/ (a uvular trembling), /l/ to /v/, /Z/ to /z/, and /S/ to /s/ were specific characteristics of orphans’ speech.

## 4. Conclusions and Discussion

The differences in speech behavior strategies of mothers during interaction with TD children, children with ASD, and children with DS were revealed. The strategies of mothers’ speech behaviors leading to the progress of the speech development of TD children were defined. It was shown that mothers of children with ASD adapted their speech to the level of the child’s speech development and were guided by the degree of the autistic disorders of the child, but this does not always lead to progress in the child’s speech development. The speech of mothers of DS children was characterized by clearer articulation of stressed vowels in words and longer duration of pauses between phrases in the utterance than the speech of mothers of TD children. Our data, in general, corresponds to the results obtained on Italian mother–child dyads of children with the developmental age of two years and different chronological ages [10]. Comparing “mother–child” dyads with older children, we revealed the differences between the speech strategies of ASD and DS children’s mothers. The speech behavior strategies of mothers of children with DS were more connected with the emotional manifestation in the child’s behavior. Mothers of ASD children stimulated the child to make verbal replies. In the speech of mothers of ASD children and children with DS, there were features aimed at stimulating the speech of children, e.g., indications, repetition of words, and repetition of words after a child, i.e., MS features peculiar to mothers of TD infants [17]. The results of the study were considered in the framework of the theoretical construct of L.S. Vygotsky’s “zone of proximal development” [18]. This study highlights the strengths and weaknesses of parental communication to children with Down syndrome and helps to identify areas of potential improvement through intervention [7]. However, it was shown that properly organized MS is not enough to correct the defects that cause the specificity of child development.

There was a lack of a large number of consonant phonemes is the specific feature of DS children’s speech compared to the speech of children with ASD, TD children, and orphans. On the basis of perceptual analysis, the differences in the recognition of children’s words by listeners were revealed—the words of children with DS were recognized worst and the words of TD children were recognized with maximal probability. The words of children with ASD and orphans were recognized with medium probability. Our results showed a lack of input leading to violations in speech acquisition in orphans that corresponds to the data of other studies [13]. At the same time, the speech and nonverbal behavior of orphans with DD and ID during interactions with adults were closer to the speech and nonverbal behavior of TD children than children with ASD and DS.

The strategies of mothers’ speech behaviors during interactions with ASD and DS children described in the work are useful for working with parents of children with atypical development. The features of a mother’s behavior associated with a high level of speech development in the child can be used for training staff working with children of preschool age with atypical development.
